# Genistein acts as antidepressant agent against chronic mild stress‐induced depression model of rats through augmentation of brain‐derived neurotrophic factor

**DOI:** 10.1002/brb3.2300

**Published:** 2021-08-01

**Authors:** Mingxiu Chang, Li Zhang, Huiyu Dai, Ling Sun

**Affiliations:** ^1^ The Fourth Affiliated Hospital of Harbin Medical University Harbin Heilongjiang China

**Keywords:** antidepressant, brain‐derived neurotrophic factor, genistein, monoamines, sucrose

## Abstract

In this study, the antidepression effects of genistein were investigated in rats induced with chronic mild stress. Animals were designated into the following groups: normal control, control, 10 mg, and 100 mg. The dose was given for 45 consecutive days via the oral route. Sucrose preference analysis, forced swim, and open field tests were performed, and serum cortisol and monoamine levels in brain tissue were determined. The mRNA and protein expression of brain‐derived neurotrophic factor (BDNF) was also examined. Supplementation with genistein significantly increased the sucrose preference ratio, locomotor activity, and monoamines and decreased serum cortisol levels. The mRNA expression of BDNF in the brain tissue was substantially reduced by 0.73% in control rats. However, supplementation with genistein significantly increased BDNF mRNA expression (by 107% and 229.6% in groups 10 mg and 100 mg, respectively). Similarly, the protein expression of BDNF increased by 82.3% and 141.2% in groups 10 mg and 100 mg, respectively. Taken together, these results suggest that supplementation with genistein may be effective against depression.

## Graphical abstract

Experimental results suggest that supplementation with genistein may be effective against depression.

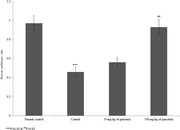

## 1 INTRODUCTION

Depression is a state of aversion to activity and low mood, and affects behavior, thoughts, feelings, sense of well‐being, and tendencies (Planchez et al., 2019). Depression is a chronic, recurring, and severe life‐threatening illness that affects people globally (Du et al., 2020). It can also be a side effect of medical treatments and physical exercise or a symptom of dysthymia (Rahmati et al., 2017). Freitas et al. (2013) have reported that the monoamine oxidase A, tricyclic antidepressants, and specific serotonin and noradrenaline reuptake inhibitors are medically available drugs for the treatment of depression. However, weight gain, sleep disorder, sexual dysfunction, cardiac toxicity and hypokinesia are main side effects of these drugs (Jiang et al., 2017). Therefore, potential agents are required for the treatment of depression without any main side effects.

Genistein is trihydroxyisoflavone found in soybeans and known to have estrogenic activity (Shen et al., 2018). Genistein has been extensively investigated due to its effects on osteoporosis, cancer, cardiovascular diseases, and depression (Atteritano et al., 2014; Kageyama et al., 2010; Marini et, al.,2010). Researchers have recommended the genistein as remedy for the menopausal symptoms and osteoporosis (Dixon & Ferreira, 2002). Researchers have reported that antidepressant‐activity through reduction of immobility time in forced swimming test (Sapronov & Kasakova, 2008), and similar effect was observed in ovariectomized female rats, suggesting the strong antidepressant‐activity of genistein. However, the mechanism of action of genistein against depression is yet to be investigated. Thus, the present study evaluated the effect of genistein on chronic mild stress‐induced depression in male albino rats.

## 2 MATERIALS AND METHODS

### 2.1 Rats

Male albino Wistar rats (weight: 180–210 g; age: 3–4 months) were purchased from the Fourth Affiliated Hospital of Harbin Medical University. The rats were maintained in standard rat polypropylene cages (435×290×150 mm; six rats in each cage) and maintained under standard atmospheric conditions of 12 h of light and dark periods with a relative humidity of 60 ± 5% and temperature of 25 ± 0.5°C with food and water provided ad libitum. All the animal experiments were approved (Ref: 2021/2T×1321) by the ethical committee of Fourth Affiliated Hospital of Harbin Medical University, Harbin, Heilongjiang, China.

### 2.2 Induction of chronic mild stress

Chronic mild stress was induced in rats according to a previously described procedure with slight modifications (Willner, 2016). Briefly, animals were trained to consume 1% sucrose solution before applying stress. Chronic mild stress protocols contain several unpredictable mild stressors, such as one period of tilted cage (3 h), one period of shaking (15 min), one period of exposure to empty bottle (1 h), one period of continuous light (36 h), one period of paired caging (2 h), one period of wet cage (21 h), and one period of water and food deprivation. The procedure was repeated for 28 days.

### 2.3 Groups and treatments

Rats were designated into four groups: normal control, control, 10 mg/kg of genistein, and 100 mg/kg of genistein. The dose was given for 45 consecutive days via the oral route. Each group contained six rats.

### 2.4 Sucrose preference analysis

The sucrose preference test was performed as previously described (He et al., 2020). Briefly, the two bottles of sucrose solution (1%) were placed on the rat cage separately. Then, free access was provided to the rats to drink water from these cages for 1 day. Then, one bottle was continued with the same sucrose solution and another bottle was filled with water for the next 24 h. Bottle position was changed to avoid the influence of bottle position. Then, all the animals were deprived of water for another 23 h, and sucrose preference examination was performed for each rat. Then, the amount of water and sucrose solution consumed was recorded and calculated.

### 2.5 Behavioral test

Forced swim and open field tests were performed as previously described (Valencia et al., 2019). All the rats were placed in the center of the open field (square chamber [80 cm], high walls [40 cm], and light [80 lux]) for 180 s in a silent room following rat weighed. Times of rearing and number of crossing squares were recorded.

### 2.6 Determination of serum cortisol

The serum cortisol level was estimated using an enzyme‐linked immunosorbent assay (ab108665, Abcam, Cambridge, UK). At the end of the treatment, rats were dissected and blood was collected and processed with the serum for the determination of cortisol level.

### 2.7 Determination of monoamine

The level of monoamines (i.e., 5‐hydroxytryptamine [5‐HT], noradrenaline, and 5‐hydroxyindoleacetic acid [5‐HIAA]) in brain tissue homogenate was determined as previously described (Huang et al., 2019). Briefly, brain tissue was surgically removed and homogenized. The clear supernatant was collected, and monoamine levels were determined by using a radioimmunoassay kit (Abcam).

### 2.8 RT‐PCR

The total RNA was extracted from the homogenates of brain tissues. Then, RNA pellets were washed in diethyl pyrocarbonate‐treated water; gel electrophoresis was carried out to check RNA integrity. The RNA purity was calculated by measuring at 260 nm. Total RNA was transcribed into cDNA by adding 0.5 ng of oligo dT primer and 1 μg of total RNA in a thermal cycler for 10 min at 65°C. Finally, 5× RT buffer (4 μl), 10 mM dNTPs (2 μl), and reverse transcriptase (100 U) were added to PCR tubes and incubated in a thermal cycler for 60 min at 37°C and then for 10 min at 90°C. Brain‐derived neurotrophic factor (BDNF) mRNA expression was determined by RT‐PCR (Table 1). The relative ratios of BDNF mRNA expression were determined according to the 2^–∆∆^ *^C^* ^T^ method (Mokhtari et al., 2017).

**TABLE 1:** List of primers used in RT‐PCR reaction for the amplification of brain‐derived neurotrophic factor

S.NO	Markers	Forward primer	Reverse primer
1	GAPDH	5′‐TCCCTCAAGATTGTCAGCAA‐3′	5′‐AGATCCACAACGGATACATT‐3′
2	BDNF	5′‐TGCAGGGGCATAGACAAAAGG‐3′	5′‐CTTATGAATCGCCAGCCAATTCTC‐3′

### 2.9 Immunofluorescence

The brain was dissected and sectioned. Then, sections were fixed in formalin and embedded in paraffin. Then, the sections were deparaffinized, rehydrated with xylene with graded alcohol series. The 3% hydrogen peroxide was used to inhibit endogenous peroxidase activity. Bovine serum albumin (2%) was used to block nonspecific binding sites. Brain tissue was treated with an anti‐BDNF (1:300 dilutions; ab226843; Abcam) antibody overnight and then incubated with FITC‐goat anti‐rat antibody (1:500 dilutions; ab6840; Abcam) for 1 h (Mokhtari et al., 2017). Samples were analyzed under a confocal microscope to determine BDNF expression.

### 2.10 Statistical analysis

Data are presented as the mean ± standard deviation. The means were compared using one‐way analysis of variance (ANOVA), followed by Tukey's post hoc test. Differences were considered significant at *p* < .05.

## 3 RESULTS

**FIGURE 1:**
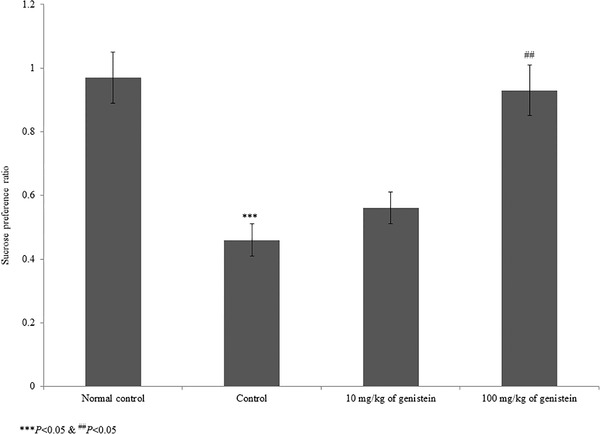
Protective effect of genistein on the sucrose preference test in an experimental model of chronic mild stress‐induced depression. ***p< .001 versus normal control and ^##^ *p*< .01 versus control

The present study evaluated the protective effect of genistein on chronic mild stress‐induced depression. Figure 1 shows the sucrose preference ratio of control and genistein‐treated rats. The sucrose preference ratio was substantially reduced by 110.9% in control rats compared to normal control rats. However, supplementation with genistein significantly increased the sucrose preference ratio to 21.7% and 102.2% at 10 and 100 mg/kg of genistein, respectively (Figure 1, *p* < .05).

**FIGURE 2:**
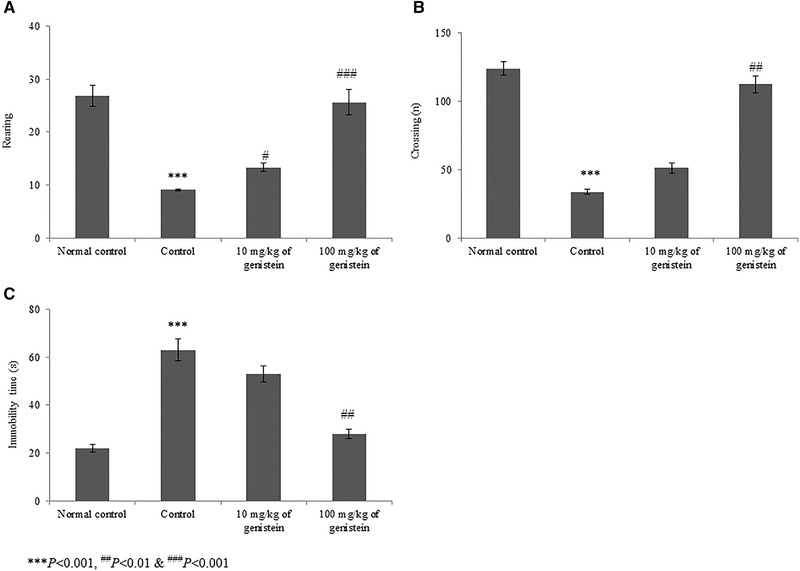
Genistein improves the behavioral parameters in an experimental model of chronic mild stress‐induced depression. (a) Represents the rearing counts, (b) represents the crossing counts, and (c) represents the immobility time. ***p < .001 versus normal control, ^#^ *p*< .05, ^##^ *p*< .01, and ^###^ *p*< .001 versus control

Behavioral parameters, such as rearing, crossing, and immobility time, were determined in control and genistein‐treated rats. Rearing capacity was substantially reduced by 66.1% in the control rats. However, supplementation with genistein significantly increased rearing by 46.2% and 181.3% at 10 and 100 mg/kg of genistein, respectively (Figure 2a, *p* < .05). Crossing counts were substantially reduced by 72.8% in the control rats. However, supplementation with genistein significantly increased crossing counts by 51.5% and 232.8% at 10 and 100 mg/kg of genistein, respectively (Figure 2b, *p* < .05). Immobility time was substantially increased by 186.3% in control rats. However, supplementation with genistein significantly reduced immobility time by 15.9% and 55.5% at 10 and 100 mg/kg of genistein, respectively (Figure 2c, *p* < .05).

**FIGURE 3:**
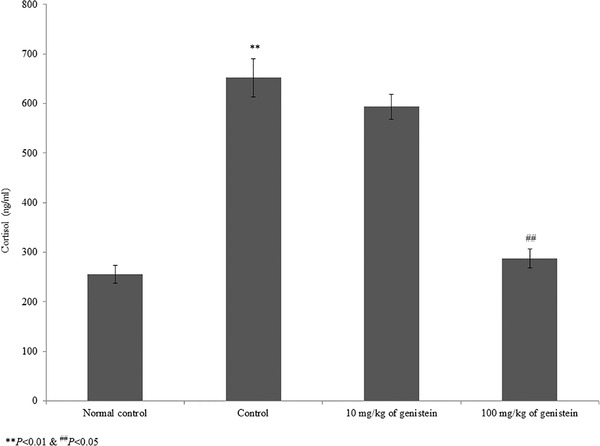
Genistein decreases the serum cortisol in an experimental model of chronic mild stress‐induced depression. **p< .01 versus normal control and ^##^ *p* < .01 versus control

**FIGURE 4:**
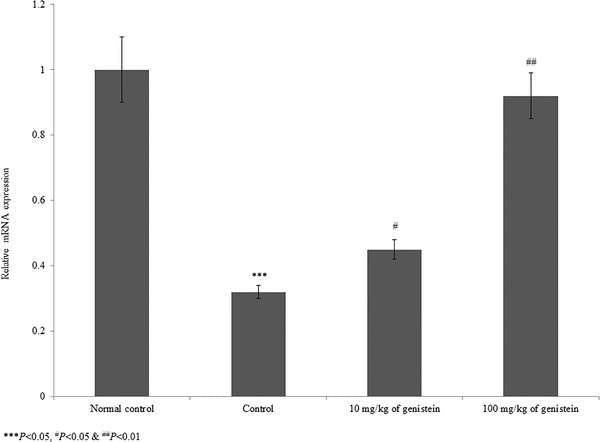
Genistein increases the brain‐derived neurotrophic factor (BDNF) mRNA expression in an experimental model of chronic mild stress‐induced depression. ***p< .001 versus normal control, ^#^ *p*< .05 and ^##^ *p* < .01 versus control

**FIGURE 5:**
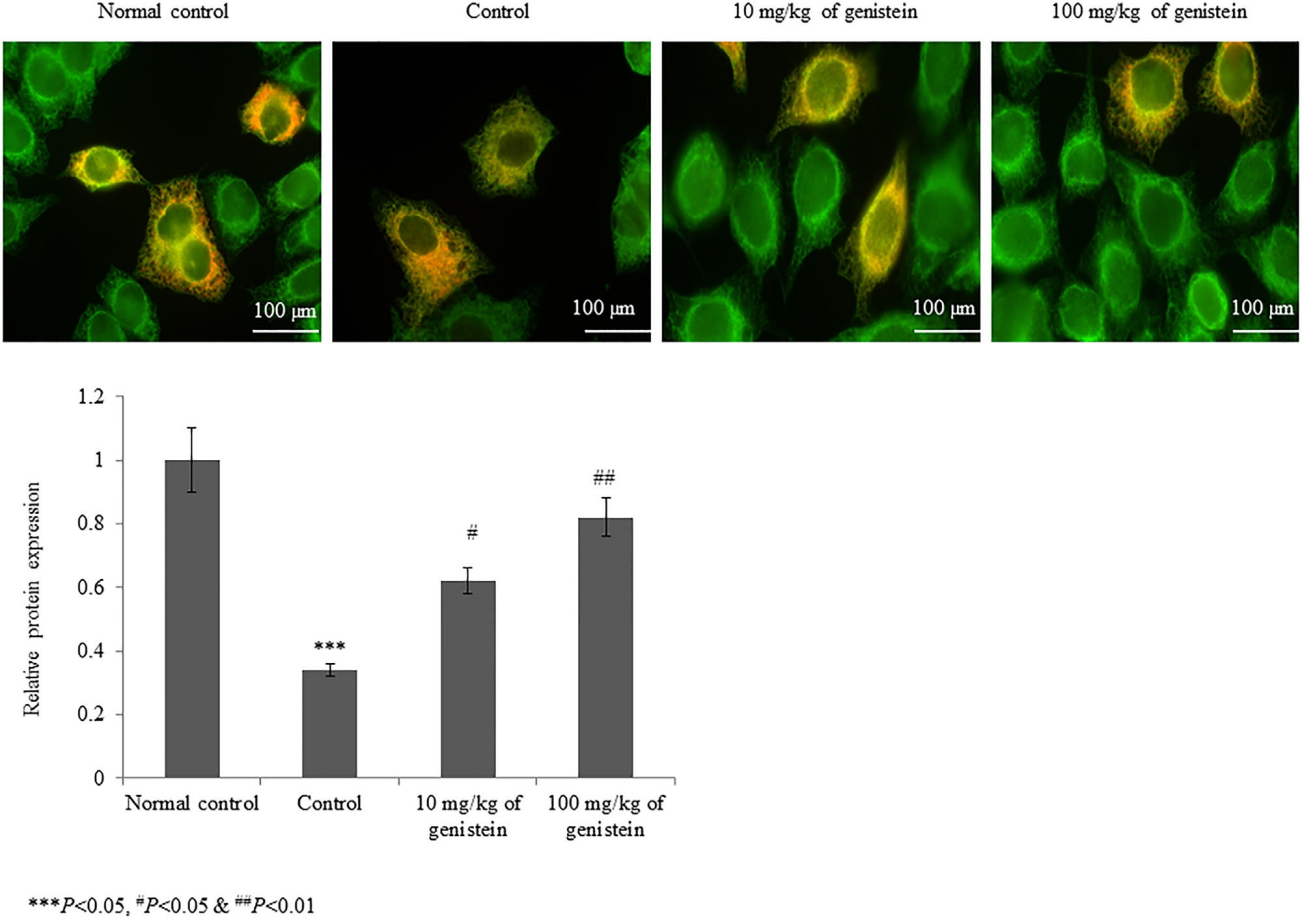
Genistein increases the brain‐derived neurotrophic factor (BDNF) protein expression in an experimental model of chronic mild stress‐induced depression. (a) Represents the microscopical images of BDNF expression, and (b) represents the quantitative estimation of BDNF expression. ***p< .001 versus normal control, ^#^ *p*< .05 and ^##^ *p* < .01 versus control

The serum cortisol level was substantially increased by 155.2% in the control rats. However, supplementation with genistein significantly reduced the cortisol level by 9% and 55.9% at 10 and 100 mg/kg of genistein, respectively (Figure 3, *p*< .05). The level of monoamines (e.g., 5‐HT, noradrenaline, and 5‐HIAA) was substantially reduced in brain tissue homogenate. However, supplementation with genistein significantly increased these monoamine levels to near‐normal levels (Table 2, *p*< .05). In brain tissue, BDNF mRNA was substantially reduced by 68% in the control rats. However, supplementation with genistein significantly increased BDNF mRNA expression by 40.6% and 187.5% at 10 and 100 mg/kg of genistein, respectively (Figure 4, *p*< .05). Similarly, the protein expression of BDNF was increased by 82.3% and 141.2% at 10 and 100 mg/kg of genistein, respectively (Figure 5, *p*< .05).

**TABLE 2:** Effect of genistein on monoamine levels in chronic mild stress‐induced rats

Monoamines	Normal control	Control	10 mg/kg of genistein	100 mg/kg of genistein
5‐HT (ng/g)	291.5 ± 13.6	54.2 ± 3.1^***^	147.4 ± 8.5^#^	266.6 ± 11.5^##^
Noradrenaline (ng/g)	137.7 ± 7.5	34.3 ± 2.5^***^	77.9 ± 4.2^#^	121.5 ± 8.2^###^
5‐HIAA (ng/g)	274.3 ± 12.8	63.5 ± 3.3^***^	144.3 ± 8.4^#^	245.1 ± 10.8^###^

^***^p<.05.

^#^*p*<.05.

^##^*p*<.01.

^###^*p*<.001.

## 4 DISCUSSION

The present study evaluated the protective effect of genistein on chronic mild stress‐induced depression. Plants and plant‐derived agents have recently attracted the attention of researchers for their therapeutic effects against several illnesses, including mental disorders (Ekor, 2014). Researchers have reported that various herbal plants and herbal formulations are useful against experimental depression (Jawaid et al., 2011). Genistein has been extensively investigated due to its effects on osteoporosis, cancer, cardiovascular diseases, and depression (Atteritano et al., 2014; Kageyama et al., 2010; Marini et al., 2010). Researchers have recommended the genistein as remedy for the menopausal symptoms and osteoporosis (Dixon & Ferreira, 2002). Researchers have reported that antidepressant‐activity through reduction of immobility time in forced swimming test (Sapronov & Kasakova, 2008), and similar effect was observed in ovariectomized female rats, suggesting the strong antidepressant‐activity of genistein.

In the present study, we analyzed the antidepressant effect of genistein on chronic mild stress‐induced depression in a rat model. Supplementation with genistein significantly increased the sucrose preference ratio, locomotor activity, monoamine levels, and BDNF expression and decreased the serum cortisol level, indicating the antidepressant activity of genistein. Chronic mild stress is widely used for the induction of an experimental model of depression in rodents and to screen for antidepressants (Hu et al., 2010). Gaurav et al. (2015) have reported that the antidepressant activity of genistein with amitriptyline. Hu et al. (2017) have reported the antidepressant activity of genistein in mice through serotonergic system.

 Researchers have reported that an increased level of serum cortisol leads to severe behavioral alterations, such as depression (Busquet et al., 2010). In the current study, chronic mild stress increased serum cortisol. This result suggested that the behavioral alterations were due to an increased cortisol level. Supplementation with genistein decreased the serum cortisol level, indicating the protective effect of genistein against depression. Monoamines, such as 5‐HT, noradrenaline, and 5‐HIAA, were reduced in the chronic stress‐induced depression model. Supplementation with genistein significantly increased these monoamines to near‐normal levels, indicating the protective effect of genistein against depression. The mRNA and protein expression of BDNF was significantly reduced in the chronic stress‐induced depression model. However, supplementation with genistein significantly increased BDNF mRNA and protein expression. Jiang et al. (2017) have reported that the genistein decreases isoflurane‐induced neurotoxicity and improves impaired memory and spatial learning by regulating the expression of BDNF‐TrkB‐PI3K/Akt. Our results agree with previous reports indicating that supplementation with curcumin increases BDNF expression and exhibits antidepressant activity (Hurley et al., 2013). Taken together, these results suggest that supplementation with genistein can be useful against depression.

## COMPETING INTERESTS

All authors declare that they have no conflict of interests.

## ETHICS APPROVAL AND CONSENT TO PARTICIPATE

All the animal experiments were approved (Ref: 2021/2T×1321) by the ethical committee of Fourth Affiliated Hospital of Harbin Medical University, Harbin, Heilongjiang, China.

## CONSENT FOR PUBLICATION

This article does not contain any studies involving human participants performed by any of the authors.

## AUTHORS’ CONTRIBUTIONS

MC conceived and designed the research. LZ and HD performed experiments and statistical analysis. LS wrote the manuscript. All authors read and approved the final manuscript.

## AVAILABILITY OF DATA AND MATERIALS

The data and material during the current study are available from the corresponding author on reasonable request.
